# Sagittal plane articulation of the contralateral knee of subjects with posterior cruciate ligament deficiency: an observational study

**DOI:** 10.1186/1749-799X-7-12

**Published:** 2012-03-20

**Authors:** Sivashankar Chandrasekaran, Jennifer M Scarvell, Graham Buirski, Kevin R Woods, Paul N Smith

**Affiliations:** 1 Trauma and Orthopaedic Research Unit, The Canberra Hospital, PO Box 11., Woden ACT 2606, Australia; 2 Department of Radiology, The Canberra Hospital, Canberra, Australia

**Keywords:** Posterior cruciate ligament, Posterior cruciate ligament injury, Knee articulation, Contralateral knee in posterior cruciate ligament injury, Risk factors for posterior ligament injury

## Abstract

**Background:**

The aim of the present study was to compare the in vivo articulation of the healthy knee to the contralateral knee of subjects with acute and chronic PCL injuries.

**Methods:**

Magnetic resonance was used to generate sagittal images of 10 healthy knees and 10 knees with isolated PCL injuries (5 acute and 5 chronic). The subjects performed a supine leg press against a 150 N load. Images were generated at 15 degree intervals as the knee flexed from 0 to 90 degrees. The tibiofemoral contact (TFC), and the centre of the femoral condyle (as defined by the flexion facet centre (FFC)), were measured from the posterior tibial cortex.

**Results:**

There was no significant difference in the TFC and FFC between the healthy knee and contralateral knee of subjects with acute and chronic PCL injuries in the medial and lateral compartments of the knee.

**Conclusions:**

The findings of this study suggest there is no predisposing articulation abnormality to PCL injury, in the setting of chronic injury the contralateral knee does not modify its articulation profile and the contralateral knee can be used as a valid control when evaluating the articulation of the PCL deficient knee.

## Background

Cadaveric studies demonstrate that the posterior cruciate ligament (PCL) is the most important constraint to posterior translation of the tibia above 30 degrees of knee flexion[1,2]. This finding has been supported by invivo studies that have demonstrated significant posterior translation of the medial tibia in subjects with PCL injuries as the knee flexes from 0 to 90 degrees in comparison to the contralateral side[3]. However, it is debateable whether the contralateral knee can be used as a valid normal control because anatomical variations such as narrower intercondylar notch anatomy and variation in tibial slope have been identified as risk factors for cruciate ligament injury[4]. The cruciate ligaments provide important proprioceptive feedback about knee stability[5]. It has not been investigated whether the contralateral knee in a subject with a PCL injury undergoes any adaptive changes as a result of the abnormal articulation pattern in the injured knee. The aim of this study is to investigate whether the contralateral knee in subjects with acute and chronic PCL injuries can be used as a valid healthy control and whether the articulation pattern in the contralateral knee of chronic PCL deficient subjects exhibits adaptive articulation patterns.

## Methods

### Study design

This is a case control study that uses a MRI model, previously described to study ACL injuries[6-9], to study the sagittal plane articulation of the tibiofemoral joint of the contralateral knee of subjects with PCL deficiency.

### Subject selection

Twenty subjects participated in the study. 10 subjects with no history of knee complaints and normal clinical examination were used as controls. The control subjects were aged between 26 and 39 years. There were 5 females and 5 male subjects. 10 subjects with unilateral PCL injuries were recruited for the study. Isolated PCL injury was diagnosed on clinical examination and MRI. On clinical examination PCL injury was suggested by posterior sag and posterior draw test. The dial test was used to exclude subjects with concomitant posterolateral corner injuries. Subjects were excluded if there were any contraindications to MRI, may have been pregnant, or if they were over 180 cm tall (to permit knee flexion in the MRI tunnel). 5 subjects had an acute PCL injury and 5 subjects had a chronic PCL injury. An acute PCL injury was defined as the persistence of bone bruising on diagnostic MRI at the time the study was undertaken. At the time the study was undertaken, the time since injury ranged from 2 to 9 weeks. There were 2 females and 3 males. The age of these subjects ranged from 18 to 27 years. 4 of the subjects had a knee brace locked in extension and had not began physiotherapy exercises and one subject was 3 weeks from removal of the brace and had begun quadriceps strengthening exercises. All subjects had an effusion on examination, decreased range of motion compared to the contralateral knee but no patellofemoral crepitus. Four subjects sustained the injury through sports (one from netball, one from soccer and two from rugby) and one subject outside of sport (sustained injury whilst falling from a two metre height). The age of the subjects with a chronic PCL injury ranged from 39 to 47 years. There were 3 females and 2 males. PCL injuries were sustained from a time period of 5 to 21 years. No subject had bone bruising on MRI at the time of the study and no effusion on clinical examination. All subjects complained of no symptoms from their knee during activities of daily living. All subjects were able to bicycle with their knee injury. All subjects in the chronic PCL group sustained the injury whilst playing sport (two from rugby and three from netball). Clinically the chronic PCL injury group were examined for evidence of degenerative joint disease. None of the subjects demonstrated joint line tenderness or had reduced range of motion compared to the contralateral side. However, all of the subjects had patellofemoral joint crepitus. All subjects provided informed consent. Ethics approval for the study was obtained from the Department of Health and university human research ethics committees.

### MRI imaging procedure

**Figure 1:**
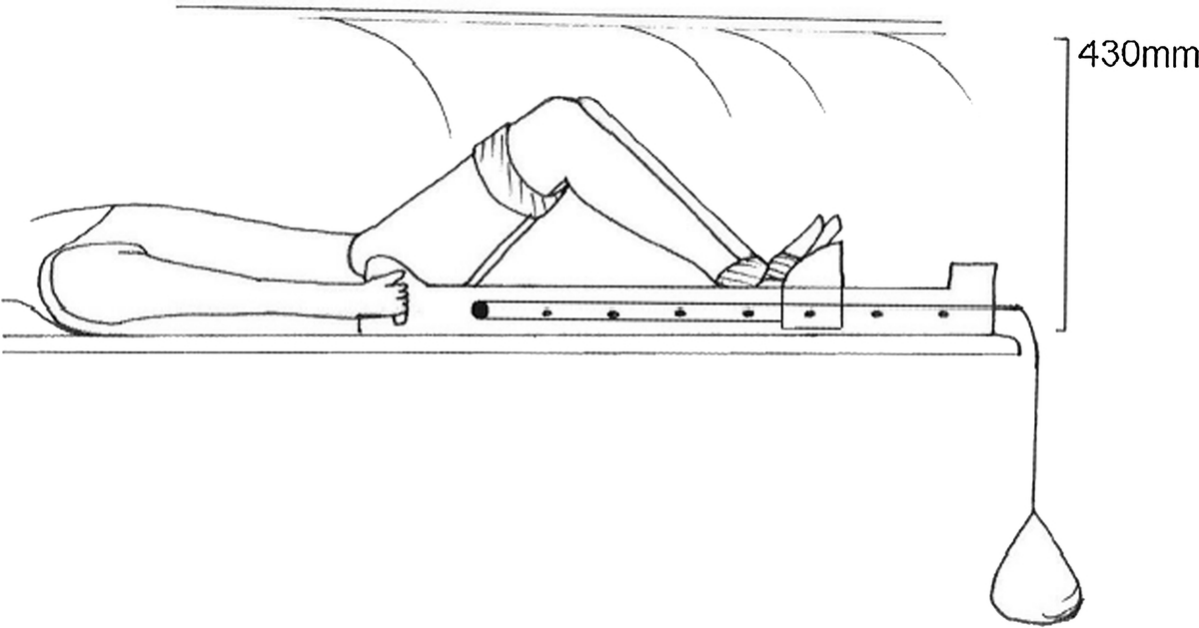
**Subjects' position in the MRI scanning tunnel**. The knees were positioned at 15 degree intervals between 0 and 90 degrees flexion, pressing down through the feet against a 150 N load

Subjects performed a supine leg press between 0 and 90 degrees on a wooden frame with a sliding footplate fitted to the MRI couch. The leg press was weighted by a 150 N load via a rope and pulley to resist leg extension and thereby simulate a weight bearing squat (Figure 1). Elastic straps stabilised the thighs, feet and ankles. Imaging of both knees simultaneously was performed. Parasagittal images perpendicular to the tibial plateau were generated through each knee

### Tibiofemoral contact point measurement

**Figure 2:**
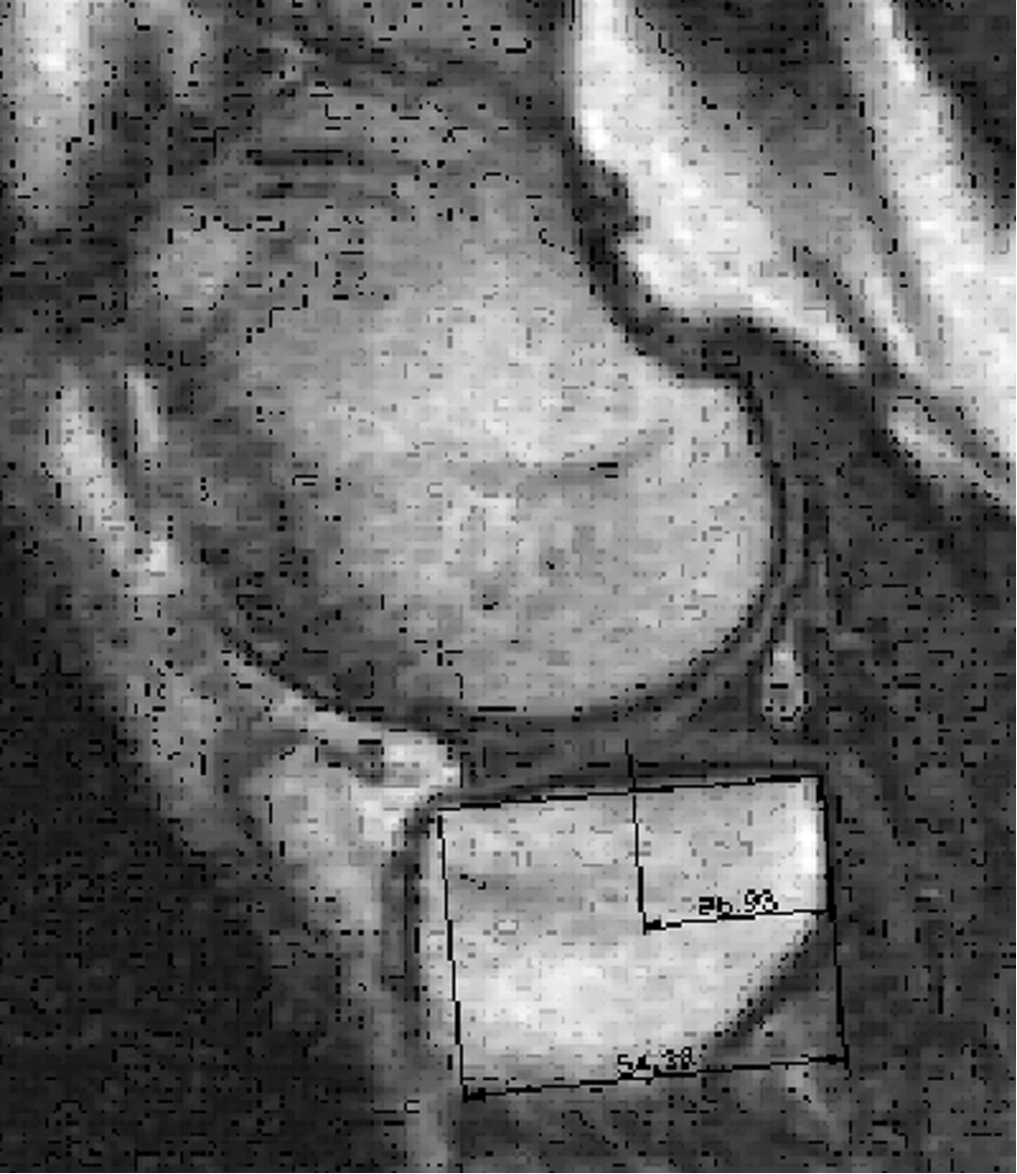
**Sagittal images through the centre of the compartment of the knee were used to measure the tibiofemoral contact**. The distance was measured through the posterior tibial cortex to the centre of the area of contact

The position of the tibiofemoral contact (TFC) with the tibial plateau was recorded as the distance from the posterior tibial cortex to the point of the TFC of the medial and lateral femoral condyle (Figure 2). Where contact occurred over a wide area, the area centroid was used. To account for variation in the size of subjects, cortex to contact distance measurements were normalised to a tibial plateau size of 50 mm. The mean anterior-posterior diameter of the medial tibial plateau was 48 +/- 5.4 mm, and the lateral tibial plateau was 41 +/- 2.4 mm.

### Flexion facet centre measurement

**Figure 3:**
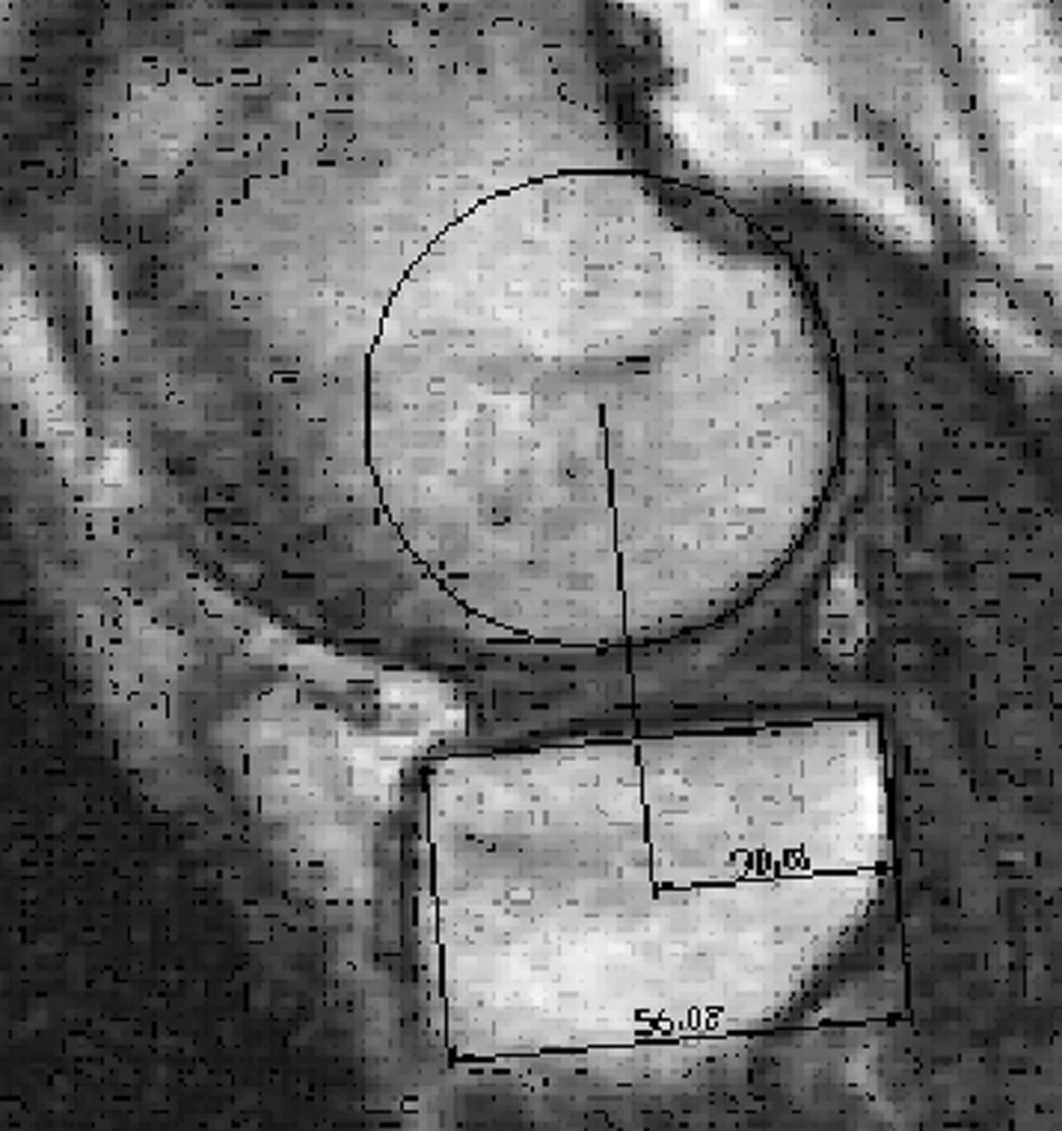
**The position of the flexion facet centre over the tibial plateau was measured in three steps: the arc and centre (FFC) of the posterior femoral condyle were defined, the tibial plateau was defined, and the distance from the perpendicular through the centre to the posterior tibial cortex was determined**.

The position of the flexion facet centre (FFC) over the tibial plateau was located by using a three stage measurement technique with a computer assisted design program (Figure 3). First, the FFC was identified by fitting a circle to define the flexion arc of the posterior condyle. This involved using an arc function to identify 3 points on the posterior aspect of the femur which could then be incorporated into a circle of bit fit. Second, the tibial plateau was defined by a line from the posterior tibial cortex, parallel to the tibial plateau. Lastly, a line was drawn through the FFC perpendicular to the tibial plateau line to measure the distance from the posterior tibial cortex to the intersection of the perpendicular line.

### Precision

The precision of both methods of measurement was tested by repeating measurement from the original scanned images on two occasions at least 24 hours apart. The precision of mapping the contact points for the medial and lateral compartments was very high with intra class correlation 0.95 (99% confidence interval was 0.92 0.96). The precision of measuring of the FFC was also very high with intra class correlation of 0.93(95% confidence interval was 0.88-0.93). The greatest difference observed between the repeated measurements was 0.7 mm for the mapping the TFC point and 0.9 mm for mapping the FFC.

### Statistical analysis

Statistical analysis was carried out using statistiXL version 1.8 for Microsoft Excel. A two-way repeated measures analysis of variance with Tukey and Scheffe post hoc tests were used to compare the TFC points and FFC positions between the healthy and the PCL deficient groups. A p value of less than 0.05 was regarded as statistically significant.

## Results

Table 1 shows the mean and standard deviations for the TFC points and FFC for the healthy and contralateral acute and chronic PCL injured knees.

**Table 1:** Mean and standard deviations for the TFC points and FCC for the healthy and contralateral acute and chronic PCL injured knees

Tibiofemoral Contact									Flexion Facet Centre			
	**Healthy**		**PCL Acute**		**PCL Chronic**		**Healthy**		**PCL Acute**		**PCL Chronic**	

**Lateral**	**Mean**	**SD**	**Mean**	**SD**	**Mean**	**SD**	**Mean**	**SD**	**Mean**	**SD**	**Mean**	**SD**

0	29.4	2.3	28.1	1.0	30.8	1.8	25.4	1.5	25.6	2.4	27.2	1.6

15	27.4	1.8	26.5	0.8	28.8	1.5	23.5	1.4	23.5	2.5	25.3	1.8

30	25.7	1.6	24.4	0.9	26.7	2.6	22.3	1.6	22.5	2.0	24.5	1.6

45	21.5	1.6	21.0	1.0	22.5	1.2	20.4	1.6	20.6	1.2	22.1	1.6

60	19.2	1.8	19.4	1.2	21.4	1.4	17.9	1.8	19.2	1.3	19.5	0.7

75	18.4	1.7	18.3	1.1	19.7	1.4	17.0	1.7	17.0	1.8	18.3	1.4

90	17.5	1.9	16.8	1.2	19.2	1.5	16.6	1.9	15.7	1.8	17.8	0.7

**Medial**												

0	31.2	1.3	31.1	1.0	32.1	1.4	20.9	1.3	20.4	0.9	22.1	1.3

15	29.3	1.2	29.0	0.7	30.2	1.0	22.5	1.2	21.3	0.4	22.8	0.9

30	25.9	1.3	26.3	0.8	27.7	1.9	22.1	1.3	21.8	0.7	23.1	0.3

45	24.1	1.4	24.5	0.5	25.5	1.3	21.8	1.4	21.3	0.4	22.1	0.4

60	23.4	1.4	23.9	1.4	24.5	1.0	20.5	1.4	20.5	0.4	22.0	1.1

75	21.1	1.4	21.9	1.3	22.7	1.7	20.3	1.4	19.9	1.3	21.9	1.2

90	21.0	1.4	22.9	1.1	22.9	2.4	20.7	1.4	19.5	0.6	21.0	0.4

### Tibiofemoral contact point

**Figure 4:**
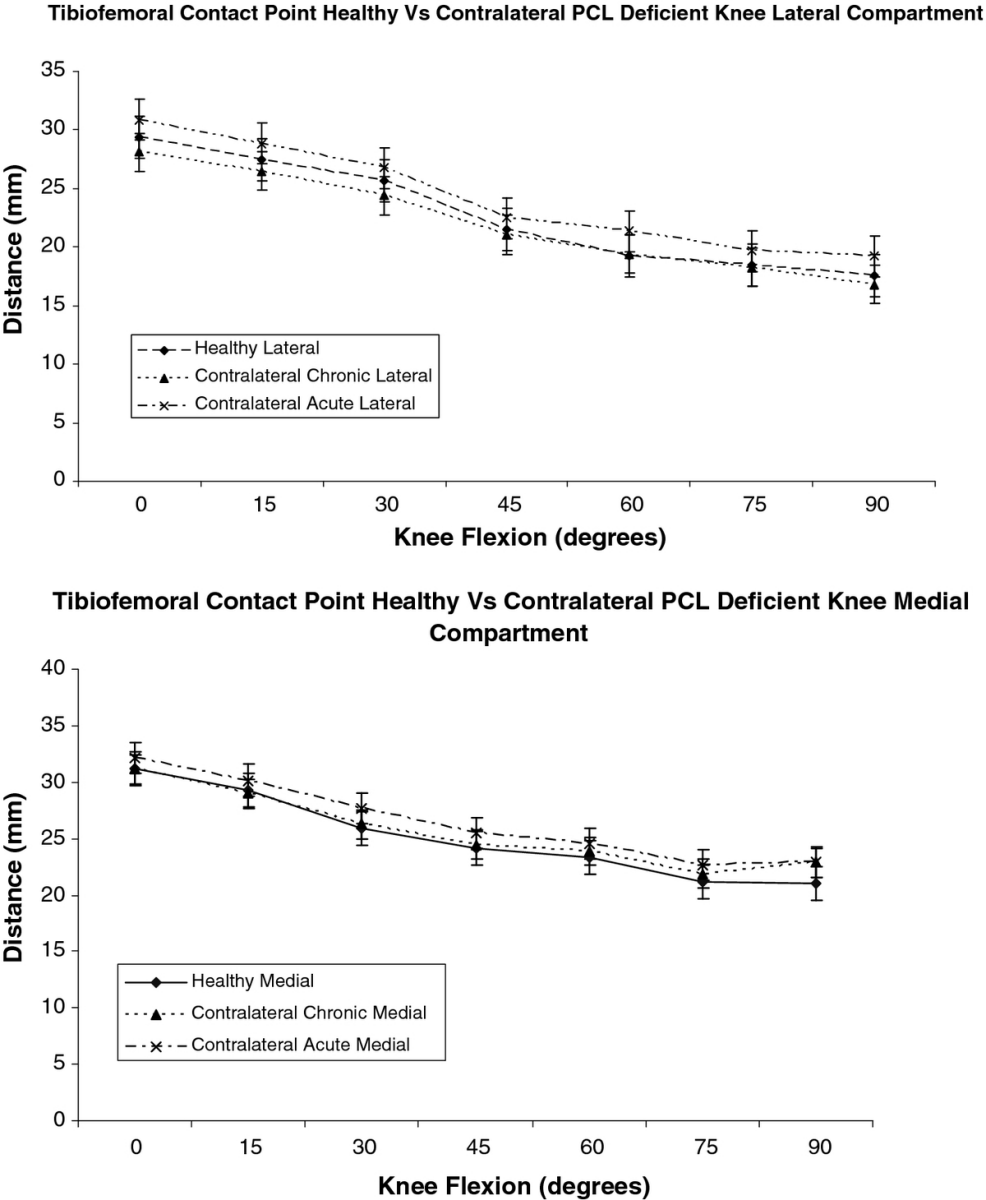
**Graph comparing tibiofemoral contact points in the healthy and contralateral acute and chronic PCL injured knees, performing a leg press against a 150 N load through a flexion arc of 0 to 90 degrees**. The pattern of tibiofemoral contact positions in healthy and contralateral acute and chronic PCL injured knees is not significantly different in both the lateral and medial compartments from 0 to 90 degrees of knee flexion

In the healthy knee the mean TFC point moved anterior to posterior as the knee flexed from 0 to 90 degrees. In full knee extension the medial compartment had a more anterior mean contact point than the lateral compartment. Between 0 and 30 degrees the mean contact point in the medial compartment moved posteriorly by 4.8 mm, which was 0.2 mm per degree. Between 0 and 30 degrees the mean contact point in the lateral compartment moved posteriorly by 3.5 mm, which was 0.1 mm per degree. Between 45 and 90 degrees the mean contact point in the medial compartment did not move posteriorly as much, 3.5 mm in 45 degrees, or 0.1 mm per degree. The mean contact point in the lateral compartment also did not move posteriorly as much 3.4 mm in 45 degrees, or 0.1 mm per degree. In the contralateral knee of subjects with an acute PCL injury for any of the mean TFC points in both the medial and lateral compartments from 0 to 90 degrees of knee flexion there was no statistically significant difference when compared to the healthy and contralateral knee of subjects with a chronic PCL injury (p > 0.05 at all TFC points). Graphically, the articulation profile of the mean TFC points of all the three groups in the study was similar in both the lateral and medial compartments (Figure 4).

### Flexion facet centre

**Figure 5:**
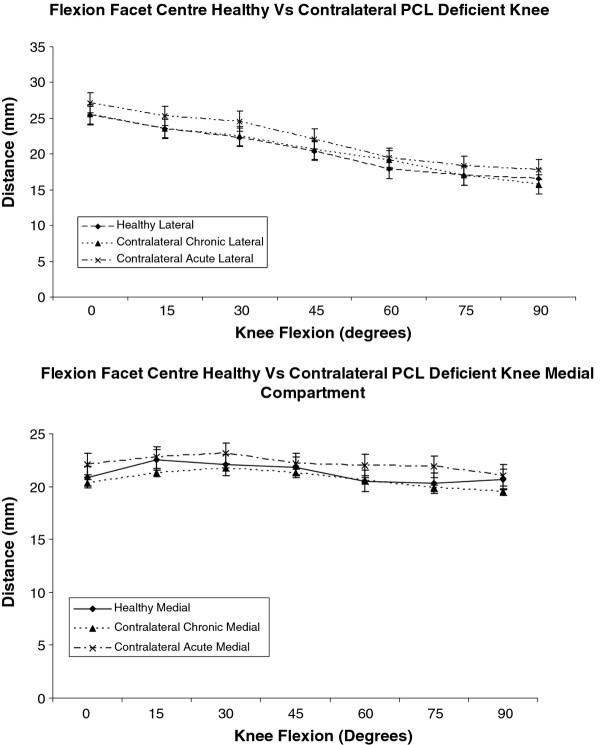
**Graph comparing flexion facet centre positions in the healthy and contralateral acute and chronic PCL injured knees, performing a leg press against a 150 N load through a flexion arc of 0 to 90 degrees**. The pattern of FFC positions in healthy and contralateral acute and chronic PCL injured knees is not significantly different in both the lateral and medial compartments from 0 to 90 degrees of knee flexion.

In the medial compartment the mean FFC was positioned posteriorly over the tibial plateau in knee extension. The medial mean FFC moved anteriorly by 2 mm as the knee flexed from 0 to 15 degrees. From 30 to 90 degrees the medial mean FFC returned to its posterior position over the medial tibial plateau. During the entire flexion arc form 0 to 90 degrees the medial mean FFC was positioned over the tibial plateau within a distance of 22.4 mm to 20.5 mm from the posterior tibial cortex. In the lateral compartment the mean FFC moved posteriorly as the knee flexed from 0 to 90 degrees. At full extension the mean FFC was located over the tibial plateau 25.3 mm from the posterior tibial cortex. At 90 degrees the mean FFC in the lateral compartment was located over the tibial plateau 16.9 mm form the posterior tibial cortex. The mean FFC in lateral compartment had moved 8.4 mm at an average of 0.1 mm per degree. In the contralateral knee of subjects with an acute PCL injury for any position of the mean FFC in both the medial and lateral compartments from 0 to 90 degrees of knee flexion there was no statistically significant difference when compared to the healthy and contralateral knee of subjects with a chronic PCL injury (p > 0.05 at all FFC positions). Graphically, the articulation profile of the mean FFC positions of all the three groups in the study was similar in both the lateral and medial compartments (Figure 5).

## Discussion

The aim of this study was to compare the sagittal plane tibiofemoral articulation of the contralateral knee in subjects with acute and chronic PCL injuries to healthy controls to determine whether it is appropriate to use the contralateral knee as a healthy control and whether the contralateral knee undergoes adaptive articulation due to altered proprioception from the injured knee. This study reported that there was no significant difference in the position of the TFC points and FFCs for both the lateral and medial compartments in the contralateral knee of subjects with acute and chronic PCL injures and healthy controls. The implications of these findings is that the contralateral knee of subjects with PCL deficiency can be used as a valid control in invivo articulation studies and there appears to be no adaptive changes in the articulation pattern of tibiofemoral joint in subjects with chronic PCL deficiency.

The limitations of this study include supine articulation, small sample size, and the cross sectional nature of the study. The supine leg press was intended to simulate a squat. It is difficult to extrapolate whether this replicates the forces during sporting or activities of daily living and as such could potentially be a source of error in our results. The reason for the small sample size is that isolated PCL injures are rare and often managed in the community with a minority referred for specialist opinion. This may explain why in vivo studies on PCL deficient articulation generally have small participant numbers[3]. The obvious limitation of the small number of study participants is the reduced power of the study and the difficulty in standardising the groups for age, sex and level of sporting activity. A prospective study that sequentially analysed the articulation of patients with a PCL injury through the acute and chronic phases would provide a more in depth analysis of temporal changes in knee articulation with a PCL injury. The difficulty with this design however, is the prolong time period for the study and potential loss of patients to follow up. Nevertheless, the authors plan to follow up the acute PCL patient group over a 2 to 5 year period to better understand the temporal changes in knee articulation with a PCL injury.

There are several studies in the literature that have examined risk factors for ligamentous knee injury. The majority of these studies have focused on anterior cruciate ligament (ACL) injuries. These risk factors can be subdivided into anatomical, neuromuscular and familial factors[4]. Anatomic factors include female gender, intercondylar notch stenosis, small ACL volume, increased posterior slope of the tibia and knee hyperextension. Neuromuscular factors include reduced proprioception, reduced quadriceps to hamstring ratio, inadequate muscle stiffness, high degree of dynamic valgus motion in landing and decreased neuromuscular control to the trunk. Although specific familial factors are difficult to isolate the literature suggests that there is at least some genetic component in the risk of sustaining an ACL injury. No studies were found that evaluated whether there was a pre-injurious abnormality in joint articulation pattern in subjects with PCL injuries. The results of this study suggest that there is no predisposing abnormal knee articulation to PCL injury.

Anatomical and biomechanical studies have shown that the ligamentous structures of the knee not only act as stabilisers but also provide proprioception[5]. In vivo articulation studies have shown that acute PCL injury produces abnormal knee articulation in the medial compartment of the knee. Specifically, there is posterior subluxation of the medial tibia with respect to the femur as the knee flexes from 0 to 90 degrees[3]. It is not known whether the contralateral knee modifies its articulation to adapt to altered proprioception from the injured knee. The results from this study show that in subjects in chronic PCL injuries the contralateral knee maintains a similar articulation profile to a healthy knee suggesting there is no adaptation. This is an important finding as abnormal articulation in the medial compartment has been associated with increased chondral and meniscal deformation forces[10].

Several studies in the literature have used the contralateral knee as a control when analysing the invivo articulation of the PCL deficient knee[3,11]. However, there have been no studies to determine whether the contralateral knee is appropriate to use as a control. The results form this study demonstrate that the contralateral knee in subjects with both acute and chronic PCL injuries does not have an altered articulation profile compared to a healthy knee and therefore can act as an appropriate control.

## Conclusions

In conclusion, this study found that there was not a significant difference in the articulation profile of the contralateral knee of subject with acute and chronic PCL injuries compared to healthy knees. This suggests that there is no predisposing articulation abnormality to PCL injury and in the setting of chronic injury the contralateral knee does not modify its articulation profile. Importantly, the contralateral knee can be used as a valid control when evaluating the articulation of the PCL deficient knee.

## Abbreviations

PCL: Posterior Cruciate Ligament; ACL: Anterior Cruciate Ligament; MRI: Magnetic Resonance Imaging; TFC: Tibial Femoral Contact; FFC: Flexion Facet Centre.

## Competing interests

The authors declare that they have no competing interests.
